# Multicenter Randomized Controlled Trial Comparing the Contribution of Proxabrushes with Regular Toothbrushes and Only Regular Toothbrushes to the Oral Health of Thai Elderly

**DOI:** 10.1155/2022/5323092

**Published:** 2022-03-31

**Authors:** Orachad Gururatana, Warangkana Vejvithee, Narisa Ekpatcha

**Affiliations:** ^1^Strategy and Quality Assurance Division, Sirindhorn College of Public Health, 29 Wachiraprakarn Road, Chonburi 20000, Thailand; ^2^Department of Health, Ministry of Public Health, Nonthaburi, Thailand

## Abstract

**Objective:**

The aim of this two-group experimental study was to explore the effects of proxabrush on Thai elderly oral health. *Design and Setting*. Multicenter randomized controlled trial, parallel grouped, open label, blocked randomization was used at each province to allocate treatment. The study was conducted at public hospitals in 16 provinces in Thailand between November 2019 and January 2020.

**Methods:**

Participants in the test group used proxabrushes and regular toothbrushes, and participants in the control group used only regular toothbrushes for 6 weeks. Plaque accumulation, gingival status, and oral healthcare data were collected at baseline and at 6-week follow-up. Clinical oral health examiner was blinded to group assignment. Randomization was computer-generated, with allocation concealment by opaque sequentially number sealed envelopes. Data analysis compared plaque and gingival indices between the test and control groups.

**Results:**

A total of 510 elderly with at least 20 natural teeth and interdental spaces were randomized (test *n* = 255; control *n* = 255), and 35 were excluded from analysis because of early drop out leaving 239 in the test group and 236 in the control group. A sample of 475 aged between 60 and 91 years participated in the study; 158 (33.3%) were males and 317 (66.7%) were females. The results revealed that, compared to baseline knowledge, attitude and practice were improved at follow-up for both the test and control groups (*p* < 0.05). At follow-up, the independent-samples *t*-test compares the test group plaque index mean of 0.49 (SD 0.44) to the control group mean of 0.60 (SD 0.56); a mean difference of 0.11 (95% CI 0.02 to 0.20) obtained demonstrated statistical significance (*p* = 0.014), and comparing the test group gingival index mean of 0.52 (SD 0.50) to the control group mean of 0.65 (SD 0.60), a mean difference of 0.13 (95% CI 0.03 to 0.23) obtained demonstrated statistical significance (*p* = 012). At follow-up, the test group had lower plaque accumulation and better gingival status than the control group. Gender, medical problems, educational level, occupation, and age were not different between the test and control groups. A combination of daily proxabrush and regular toothbrush use yielded significant benefits over regular tooth-brushing alone.

**Conclusions:**

In conclusion, proxabrush has been found to be an effective interdental cleaning aid among Thai elderly. This trial is registered with TCTR20220127004.

## 1. Introduction

Gingival disease involves enlarged gingival contour, changes in the gingival color to red or bluish-red, increased sulcular temperature, and increased gingival exudate. In severe gingival disease, gingiva may bleed on probing or spontaneously [1]. Gingival disease is considered a public health problem, because it can lead to serious consequences such as problems with chewing, biting, and swallowing in the elderly due to tooth loss [2] and affects the quality of life [3]. Data from the eighth oral health survey reported that oral health is a public health problem among Thai elderly. One-third of Thai elderly have periodontal problems and have lost 2 of 6 sextants of teeth [4]. In Thailand, 11.1 million elderly currently represent 16.7% of the total population. The proportion of the elderly population is increasing and expected to be 20 million by the year 2040, which is one-fifth of the entire population in Thailand [5].

The aetiological factor of gingival disease is dental plaque, a biofilm consisting of micro-organisms. If dental plaque is left on the teeth for more than two weeks, gingival disease will occur. Thus far, an efficient mechanical method is the best way to remove dental plaque [6]. Plaque removal in the interproximal areas is essential, because it is likely that interproximal areas are the first parts of the tooth where gingival disease occurs [7]. Regular tooth-brushing is effective in mechanically removing buccal and lingual dental plaque [8]. Due to its shape, however, a regular toothbrush cannot penetrate mesial and distal surfaces to remove plaque in interproximal areas [8].

Therefore, interproximal cleaning device is needed. Recommended elderly oral self-care includes regular tooth-brushing twice daily, use of fluoride toothpaste, interdental cleaning daily, and reduction in sugar consumption [9]. Dental floss, a well-known device for plaque removal in the interproximal areas, has been found to be difficult to use [8]. In consequence, the proportion of daily dental flossing is low [10]. Studies of proxabrush efficacy in reducing gingival inflammation and suitability for patients with spaces only are controversial; however, proxabrushes tend to be easier to use and more preferred by patients than dental floss [11]. Furthermore, interproximal bristles can fill the embrasure effectively to remove plaque in the proximal areas [12]. Therefore, proxabrush is considered a potential alternative to dental floss. Use of dental floss or proxabrush to remove plaque in the interproximal areas in Thai elderly is as low as 6% [4].

At present, no studies have reported the effects of proxabrush in Thailand. It is unknown whether a proxabrush in combination with a regular toothbrush will benefit Thai elderly or not. The aim of this two-group experimental study was to evaluate whether proxabrushes used at home in addition to regular tooth-brushing can better control plaque and improve gingival status of oral health among Thai elderly than regular tooth-brushing alone.

## 2. Methods

### 2.1. Trial Design

This was a multicenter randomized controlled trial, parallel-group, open‐label, using a block size of 2 with a 1 : 1 allocation study conducted in Thailand. Dental health personnel from 39 elderly oral health-promoting provinces in Thailand including Lampang, Nan, Phitsanulok, Chainat, Pathumthani, Singburi, Saraburi, Nakhonnayok, Suphanburi, Chanthaburi, Loei, Buriram, Sisaket, Amnatcharoen, Nakhonsithammarat, Trang, Songkhla, Chiangrai, Phrae, Tak, Kamphaengphet, Nonthaburi, Ayutthaya, Lopburi, Angthong, Nakhonpathom, Ratchaburi, Samutsakhon, Chonburi, Roi Et, Surin, Mukdahan, Ubonratchathani, Krabi, Phang Nga, Ranong, Phatthalung, Bangkok, and Samutprakan were informed of the details of the study, and participation was voluntary. In all, 17 provinces chose to participate in the study, while 22 provinces declined to participate. The 17 provinces included Lampang, Nan, Phitsanulok, Chainat, Pathumthani, Singburi, Saraburi, Nakhonnayok, Suphanburi, Chanthaburi, Loei, Buriram, Sisaket, Amnatcharoen, Nakhonsithammarat, Trang, and Songkhla.

### 2.2. Sample Size Calculation

The combined elderly population of the above 17 provinces is 2,275,316. Applying the Schlesslman formula [13] an estimated sample size of 11.6 would be required for a statistical power of 80% and an alpha of 5%. A detection of a difference of at least 30% of plaque between proxabrush with regular toothbrush and regular toothbrush only was considered clinically relevant. In the absence of equivalent study to this study, the sample size calculation was based on previous study data [8] in which there were 30% differences in the mean overtime when only regular toothbrush was used (mean plaque index, 3.09; SD 0.62) and the mean plaque index when proxabrush was used in combination with regular toothbrush (mean plaque index, 2.15; SD 0.99). It was planned to recruit 15 elderly to allow for 25% loss to follow up at each site. Therefore, each province contained a group of 30 participants; 15 were randomized to the control group, and 15 were randomized to the test group.

### 2.3. Participants

Eligible participants were above 60 years old, had no systemic bleeding disorders, had at least 20 natural teeth, had interdental spaces, could speak and understand the Thai language, had no hearing or speaking difficulty, and did not suffer from depression. The study was conducted at public hospitals in 17 provinces in Thailand between November 2019 and January 2020.

### 2.4. Randomization

Following the direction of the research team, 1 : 1 allocation to the test and control groups was undertaken at each province by dental health personnel. Random allocation sequence in a block size of 2 was generated by computer.

### 2.5. Allocation Concealment

Sequentially numbered, opaque envelopes were prepared. Letters A and B represented the test and control groups, respectively. The letters were printed, cut out, and sealed in opaque envelopes. Participant's information and name were written outside the envelope before opening it.

### 2.6. Blinding

The allocation method was not revealed to the clinical oral health examiner. Clinical oral health examiners were not informed of the oral cleaning devices participants used.

To assess knowledge, attitude, and behaviour toward oral health care, a questionnaire was constructed based on a previous questionnaire administered by a Thai national oral health survey among people aged 60–74 years. The first part of the questionnaire asked general questions on age, gender, and education, while the second part assessed knowledge, attitude, and oral healthcare practice. There were 4 questions regarding knowledge, 5 questions regarding attitude, and 6 questions regarding oral healthcare practice as characteristics of good quality toothbrush, ability to prevent tooth loss, frequency of tooth-brushing, and usage of interproximal cleaning devices. The total possible scores on the second part of the questionnaire range from 11 to 37 points. Higher scores meant more positive results toward oral health care than lower scores. The psychometric properties of the questionnaire were tested. Content validity was tested by the index of item objective congruence by three experts in the field (ranging from 0.67 to 1). Internal consistency was tested by Cronbach's alpha coefficient (0.72). Difficulty and discrimination of items testing knowledge were tested (0.17–0.63, 0.21–0.64, respectively).

Following ethical approval from the Sirindhorn College of Public Health, Chonburi province, the study was explained to potential elderly participants, who were given information sheets and informed consent forms. All elderly were assured that participation was voluntary; their responses were confidential, and they could withdraw at any time. The research team arranged a meeting with dental health personnel of the 17 provinces to inform and discuss data collection procedures. Standardization in a pilot study undertaken by two dental health personnel in 30 participants had a kappa of 0.76 and had an agreement of 90%. Toothbrushes, proxabrushes, World Health Organization probes, and questionnaires were given to the dental health personnel of the 17 provinces.

### 2.7. Primary and Secondary Outcomes

Primary outcome was plaque accumulation, and secondary outcome was gingival status. Additional analyses were made on oral healthcare data.

### 2.8. Interventions

Plaque accumulation was measured by the Silness and Löe plaque index [14], gingival status was measured by the Löe and Silness gingival index [15], and oral healthcare data were collected at baseline and 6-week follow-up by the dental health personnel in each province. Plaque accumulation and gingival status data were collected at a dental unit equipped with mouth mirrors, dental explorers, and World Health Organization probes. The dental health personnel in each province demonstrated how to use proxabrushes and regular toothbrushes in the test group and demonstrated how to use regular toothbrushes in the control group. The participants in each group practiced using the assigned cleaning devices. The participants in both groups also received scaling and polishing. The dental health personnel instructed the participants to use a regular toothbrush twice a day and a proxabrush once a day. Every two weeks during the 6-week follow-up period, the dental health personnel called each participant to motivate and remind them to use cleaning devices as they had been instructed. Questionnaires were distributed at baseline and the 6-week follow-up. Assistance was available when the elderly had reading difficulty.

### 2.9. Statistical Methods

The primary outcome was plaque accumulation, which was measured by the Silness and Löe plaque index [14]. The secondary outcome was gingival status, which was measured by the Löe and Silness gingival index [15]. Independent-samples *t*-test was used to compare plaque index [14] and gingival index [15] between the test and control groups. Paired-samples *t*-test was used to compare oral healthcare data including knowledge, attitude, and behaviour between baseline and 6-week follow-up. Chi-square test was used to compare sociodemographic characteristics between the test group and the control group.

## 3. Results

A total of 510 participants from the 17 provinces were recruited to the study. There were 30 participants in each province; 15 participants were randomly assigned to the test group, and 15 participants were assigned to the control group.

### 3.1. Recruitment

Eligible participants were recruited from November 2019. Participants used oral cleaning devices as they were assigned for 6 weeks. The dates of data collection for each province were not the same, but within the period of time suggested by the research team.

Following baseline data collection in Phitsanulok Province, Thailand was attacked by the first wave of severe acute respiratory syndrome coronavirus 2 (SARS-CoV-2). As a result, the participants in this province were not able to participate in follow-up data collection and Phitsanulok Province was excluded from the study. Therefore 16 provinces remained in the study for data analysis.

**Figure 1:**
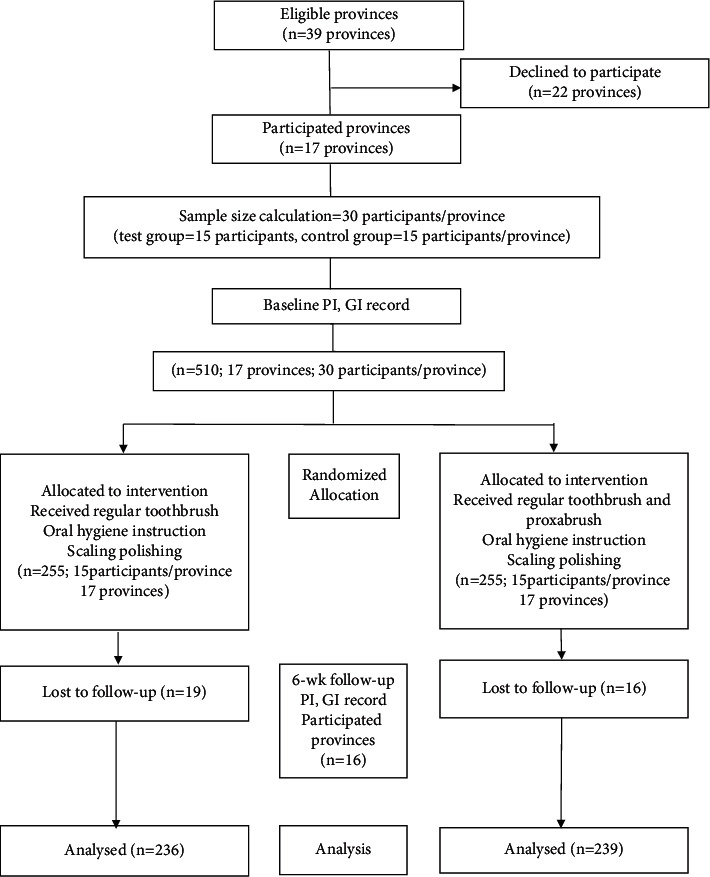
Flow diagram.

One participant from the test group and four participants in the control group tested were lost to follow up. Therefore, 239 were in the test group using regular toothbrushes and proxabrushes to clean their teeth, and 236 participants were in the control group using only regular toothbrushes for 6 weeks (Figure 1).

Sociodemographic characteristics were similar between the test group and the control group (Table 1).

Knowledge, attitude, and practice were improved at follow-up compared to baseline for both the test and control groups (Table 2).

**Figure 2:**
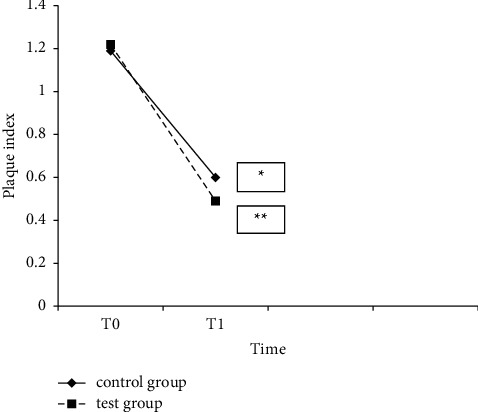
Plaque index at baseline (T0) and 6-week follow-up (T1) in the test and control groups. ^∗^*p* < 0.05 within groups. ^∗∗^*p* < 0.05 between groups.

**Figure 3:**
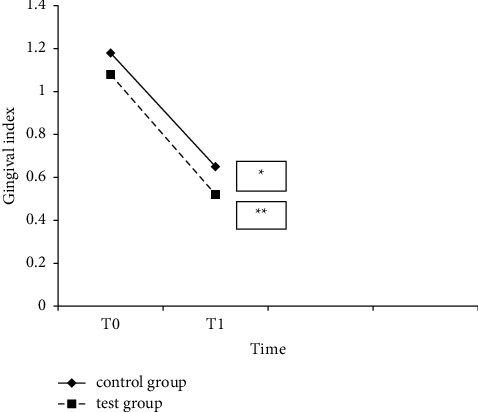
Gingival index at baseline (T0) and 6-week follow-up (T1) in the test and control groups. ^∗^*p* < 0.05 within groups. ^∗∗^*p* < 0.05 between groups.

At follow-up, the test group had lower plaque accumulation and better gingival status than the control group (Table 3, Figures 2 and 3). Routine use of a proxabrush and a regular toothbrush yielded significant benefits over regular tooth-brushing alone.

## 4. Discussion

The participants in both groups had improved knowledge, attitude, and practice at the 6-week follow-up compared to those at baseline, because dental health education was delivered by including demonstration and practice sessions. Hence, the participants in this group were able to understand better than those provided only with the instruction of dental health care. This is in line with a qualitative study finding 8 in 15 participants who received dental health education by teaching and practicing to have good in-depth understanding about how to practice good oral health care with the ability to perform the procedures in greater detail than those who only had instructions from dental health personnel or had watched videos without any practice [16]. Several researchers have reported that practicing sessions can increase confidence, self-efficacy, and changes in behaviour [17, 18]. Furthermore, periodically encouraging participants to use the oral cleaning device employed in this study twice a week can also increase the usage frequency of the device.

### 4.1. Interpretation

The difference between the test and control groups in this study was that the proxabrush was only provided in the test group. Therefore, it is possible that plaque was reduced and gingival status improved more in the test group than the control group due to the use of the proxabrush. Other studies also indicated that proxabrush in combination with a regular toothbrush is more effective in mechanical cleaning than a regular toothbrush alone [19, 20]. Mechanical plaque control using a regular toothbrush combined with a proxabrush had lower plaque and bleeding scores at a 4-week follow-up than at baseline. Furthermore, using a proxabrush demonstrated less bleeding on probing than using dental floss. There was no difference between using cetylpyridinium 0.05% gel released from a proxabrush and using proxabrush without gel. This also confirms the mechanical efficacy of proxabrushes in plaque control [20]. A study in mild to moderate periodontal patients reported similar findings as in moderate to severe periodontal patients. Tooth surfaces cleaned with a proxabrush had less bleeding on probing and periodontal pocket depth compared to baseline [21]. A comparative study between using regular toothbrushes alone and using regular toothbrushes in combination with proxabrushes reported that proximal dental plaque in the proxabrush group was lower than that in the regular toothbrush group [22].

When compared to other interdental cleaning devices, the proxabrush appears to be superior in reducing plaque, gingivitis scores, and bleeding on probing. There is a moderate level of evidence that a regular toothbrush combined with a proxabrush can reduce gingival inflammation, while there is only a low level of evidence or inconclusive evidence that a regular toothbrush combined with dental floss or a toothpick or an oral irrigator can reduce gingival inflammation. This confirms that the proxabrush yields more promising findings than the other interdental cleaning devices [23]. A study in 19 partially edentulous individuals demonstrated that a proxabrush is more effective than a toothpick in reducing plaque; however, when interdental papilla is intact, a toothpick is more effective than a proxabrush [24]. A comparative study between proxabrush and dental floss use in moderate to severe untreated periodontal patients reported as the 6-week follow-up that the proxabrush was more effective in plaque, gingival bleeding, and periodontal pocket depth reduction than dental floss [8]. In participants with interdental spaces due to periodontal loss, a proxabrush can reduce more plaque than dental floss and toothpicks [25]. A comparative study between dental floss and proxabrush use at 6-and 12-week follow-ups examining plaque, interdental papilla, gingival bleeding, probing depths, and bleeding on probing reported that the proxabrush group had better findings for all indices at 6-week follow-up than the dental floss group. At the 12-week follow-up, the proxabrush group had better findings for plaque, interdental papilla, and probing depths, although subgingival calculus had not yet been removed [26]. A comparative study among dental floss, conical, and cylindrical proxabrush use reported that both types of proxabrushes can reduce more supragingival plaque in periodontal patients than dental floss. Moreover, the findings indicated more usage frequency in the proxabrush group than in the dental floss group. This is likely because the proxabrush is easier to use than dental floss [27]. A comparative study of dental floss, flossers, proxabrushes, and soft-pick cleaners indicated that all 4 interdental devices can reduce dental plaque, but proxabrushes can reduce more buccal interdental gingival inflammation than other interdental devices. However, there was no difference in plaque reduction, gingival bleeding and gingival inflammation in the lingual interdental area. The study concluded that proxabrushes are more effective than dental floss, which is the gold standard for gingivitis reduction [28].

Although a proxabrush in combination with a regular toothbrush seems to reduce more plaque than the regular toothbrush, some researchers have stated that the effectiveness of proxabrushes in reducing gingival inflammation remains inconclusive and that it is impossible to compare gingival inflammation reduction between proxabrush and dental floss. Nonetheless, they concluded that proxabrushes can reduce more periodontal pocket than dental floss [29]. This may be due to different gingival indices selected, different follow-up periods measured, and different study designs. Therefore, more studies are needed in the future.

### 4.2. Limitations and Generalizability

There are several limitations in this study. Firstly, participants were elderly people who had interdental spaces. Therefore, generalization to elderly people without interdental spaces is limited, but most elderly have spaces between teeth due to periodontal disease. Data from the eighth national oral survey reveal that the percent of elderly without periodontal pocket is 34.7% in the group aged 60–74 years and only 17.9% in the group aged 80–85 years. Hence, it is assumed that most Thai elderly have a tendency to have interdental spaces due to the loss of periodontium and would benefit from using a proxabrush. However, several researchers suggested that proxabrushes can be used when teeth are intact [12]. Advice regarding the appropriate proxabrush size for each elderly person might be needed when teeth are intact. Secondly, the participants were from elderly oral health-promoting provinces that might have benefited from previous oral health-promoting activities at elderly oral health clubs where they might have received oral health education [30]. Therefore, generalization to nonelderly oral health-promoting provinces is limited. Finally, the study was conducted in 16 different settings and standardization of clinical oral examination was not conducted, which might have caused bias in data collection. However, the researcher team attempted to create the same standard of data collection at every setting by arranging face to face meetings with dental health personnel from all 16 settings prior to data collection to discuss and summarize data collection procedures, supplying standard dental equipment, interdental cleaning devices, and questionnaires for every setting and creating an online service to answer any queries during the data collection period. Future studies may collect data in nonelderly oral health-promoting provinces and from elderly without interdental spaces to confirm the benefits of proxabrushes.

Despite the limitations, this was the first study in Thailand to compare the use of a proxabrush and a regular toothbrush with a regular toothbrush alone and yielded encouraging findings. For each province, the dental health personnel randomized the participants into test and control groups to reduce selection bias and confounding factors. Moreover, the study included 16 from the total of 77 provinces in Thailand from all four regions across the country. Therefore, the participants can be representative of every region in the country and proxabrushes could be recommended as a potential alternative to dental floss.

The use of a proxabrush in combination with a regular toothbrush in this multicenter randomized controlled trial significantly improved plaque and gingival index scores, indicating improved oral hygiene and reduced gingival inflammation at 6-week follow-up. Therefore, proxabrush is an effective interdental cleaning aid for Thai elderly. The findings of this study suggest the promotion of proxabrush use among elderly and support for the availability of this interdental cleaning aid.

## Figures and Tables

**Table 1:** Sociodemographic characteristics at baseline.

Variables	Test (*n* = 239)	Control (*n* = 236)	*p* value
Age (mean ± SD)	66.61 ± 5.53	66.45 ± 5.58	0.757
Gender			0.436
Males, *n* (%)	75 (31.4%)	83 (35.2%)	
Females, *n* (%)	164 (68.6%)	153 (64.8%)	
Systemic disease			0.772
No systemic disease	66	69	
With systemic disease	173	167	
Education			0.198
No education	9	11	
Primary education	168	184	
Secondary education	27	21	
Certificate	13	6	
Bachelor degree/higher	22	14	
Occupation			0.096
Retired civil servant	27	13	
Merchant	12	22	
Farmer	95	106	
Unskilled worker	19	24	
No occupation	83	74	
Income			0.172
No income	21	18	
1–5000 THB/month	135	153	
>5,000 THB/month	83	65	

Age was compared using independent *t*-test. Gender, systemic disease, education, occupation, and income were compared using chi-square test. THB = Thai Baht.

**Table 2:** Mean ± standard deviation (SD) for variables measured at baseline and after 6 weeks and mean differences between baseline and follow-up scores.

	Baseline	6-week follow-up	Mean difference (95% CI of difference)	*p* value
Knowledge				
Test	2.63 ± 1.20	3.77 ± 0.54^*∗*^	−1.13 (−1.29, −0.98)	<0.001^*∗*^
Control	2.47 ± 1.20	3.32 ± 0.82^*∗*^	−0.85 (−1.01, −0.69)	<0.001^*∗*^
Attitude				
Test	12.50 ± 2.24	13.67 ± 1.95^*∗*^	−1.17 (−1.44, −0.89)	<0.001^*∗*^
Control	12.52 ± 2.21	13.86 ± 1.79^*∗*^	−1.35 (−1.62, −1.07)	<0.001^*∗*^
Behaviour				
Test	12.82 ± 2.09	16.12 ± 1.54^*∗*^	−3.30 (−3.61, −2.99)	<0.001^*∗*^
Control	12.52 ± 2.25	14.40 ± 1.72^*∗*^	−1.88 (−2.19, −1.57)	<0.001^*∗*^
Plaque index				
Test	1.22 ± 0.70	0.49 ± 0.44^*∗*^	0.73 (0.66, 0.80)	<0.001^*∗*^
Control	1.19 ± 0.68	0.60 ± 0.56^*∗*^	0.59 (0.52, 0.65)	<0.001^*∗*^
Gingival index				
Test	1.08 ± 0.67	0.52 ± 0.50^*∗*^	0.56 (0.50, 0.63)	<0.001^*∗*^
Control	1.18 ± 0.70	0.65 ± 0.60^*∗*^	0.53 (0.47, 0.60)	<0.001^*∗*^

^∗^ *p* < 0.05 paired-samples *t*-test; within groups.

**Table 3:** Comparison of differences between means for test and control groups.

Variables	Time	Mean differences (95% CI of difference)	*p* value
Knowledge	Baseline	−0.17 (−0.38, 0.05)	0.134
	Week 6	−0.44 (−0.57, −0.32)	<0.001^*∗*^
Attitude	Baseline	0.02 (−0.38, 0.42)	0.926
	Week 6	0.199 (−0.14, 0.54)	0.247
Behaviour	Baseline	−0.31 (−0.70, 0.08)	0.124
	Week 6	−1.72 (−2.02, −1.43)	<0.001^*∗*^
Plaque index	Baseline	−0.03 (−0.16, 0.09)	0.584
	Week 6	0.11 (0.02, 0.20)	0.014^*∗*^
Gingival index	Baseline	0.10 (−0.03, 0.22)	0.121
	Week 6	0.13 (0.03, 0.23)	0.012^*∗*^

^∗^ *p* < 0.05: independent samples *t*-test between groups.

## Data Availability

The data that support the findings of this study are available on request from the corresponding author. The data are not publicly available due to privacy or ethical restrictions.
